# A Particle Filtering Approach for Fault Detection and Isolation of UAV IMU Sensors: Design, Implementation and Sensitivity Analysis

**DOI:** 10.3390/s21093066

**Published:** 2021-04-28

**Authors:** Egidio D’Amato, Vito Antonio Nardi, Immacolata Notaro, Valerio Scordamaglia

**Affiliations:** 1 Dipartimento di Scienze e Tecnologie, Universitá degli Studi di Napoli “Parthenope”, 80143 Napoli, Italy; egidio.damato@uniparthenope.it; 2 Dipartimento di Ingegneria dell’Informazione, delle Infrastrutture e dell’Energia Sostenibile, Universitá degli Studi “Mediterranea” di Reggio Calabria, 89122 Reggio Calabria, Italy; valerio.scordamaglia@unirc.it; 3 Dipartimento di Ingegneria, Universitá degli Studi della Campania “L.Vanvitelli”, 81031 Aversa, Italy; immacolata.notaro@unicampania.it

**Keywords:** fault detection and isolation, particle filter, UAV fault detection, FDI, fault tolerant attitude estimation, duplex attitude estimation architecture, analytical redundancy

## Abstract

Sensor fault detection and isolation (SFDI) is a fundamental topic in unmanned aerial vehicle (UAV) development, where attitude estimation plays a key role in flight control systems and its accuracy is crucial for UAV reliability. In commercial drones with low maximum take-off weights, typical redundant architectures, based on triplex, can represent a strong limitation in UAV payload capabilities. This paper proposes an FDI algorithm for low-cost multi-rotor drones equipped with duplex sensor architecture. Here, attitude estimation involves two 9-DoF inertial measurement units (IMUs) including 3-axis accelerometers, gyroscopes and magnetometers. The SFDI algorithm is based on a particle filter approach to promptly detect and isolate IMU faulted sensors. The algorithm has been implemented on a low-cost embedded platform based on a Raspberry Pi board. Its effectiveness and robustness were proved through experimental tests involving realistic faults on a real tri-rotor aircraft. A sensitivity analysis was carried out on the main algorithm parameters in order to find a trade-off between performance, computational burden and reliability.

## 1. Introduction

In recent years the use of unmanned aircraft has significantly increased, making the mitigation of risks due to on-board avionics malfunctions essential, in view of sustainable and safe aviation.

Several applications have been exploited with military and civil objectives [1,2], including search and rescue missions in hostile environments [3], surveillance and security patrols [4], home security [5], precision agriculture [4] and network coverage [6], fields of application where UAVs play a key role in terms of reduction in operations and support costs [4].

The *flight control system* (FCS) is a key component of a generic UAV. It can include inertial sensors, air data systems, avionics, control surfaces/servos, servo-actuators, on-board software and other relevant subsystems contributing to UAV stability and control. A fault in such a system could be critical in the assessment of UAV reliability. It is worth noting that faults on FCSs account for 25% of U.S. military UAV incidents [7].

An FDI system must be able to detect and properly isolate faults in order to mitigate the effects on UAV system performance and integrity. Four key features should be present in an FDI system:

- fast detection of abnormal situations;
- isolation of faults;
- robustness to noise and uncertainties;
- low false alarm rate.

For a modern aircraft, three kinds of fault can be considered:

- sensor faults: a malfunction of the measurement subsystem;
- actuator faults: a malfunction of actuators acting on system dynamics;
- process faults: a strong change in system dynamics due to structural problems.

The most sophisticated UAVs are often equipped with technology solutions for automatically detecting and isolating faults on sensors, collectively labeled as *fault detection and isolation* (FDI) systems [8,9,10,11,12]. The presence of an FDI system increases the capability of UAVs to fulfill prescribed tasks even in the presence of faults. Quick detection and proper isolation of faults represents the first step of a reconfiguration strategy, required to minimize fault impact upon UAV performance [9,13]. In the literature, significant research efforts have been spent in terms of FDI solutions for UAVs. In [14], FDI on UAV Pitot tubes and control surfaces is carried out by using statistical change detection, while in [15], a state machine-based solution is proposed. Ref. [16] shows a sensor fusion-approach for FDI on air data sensors and [17] exploits self-tuning residual generators for fault detection on control surfaces. Finally, a cooperative FDI scheme for UAVs is proposed in [18,19] deals with the faults in UAV FDI systems.

In order to control UAV orientation with respect to an inertial reference system, one of the FCS tasks is attitude estimation. Usually, this consists of sensor fusion algorithms that use measurements coming from accelerometers, gyroscopes and magnetometers [20]. Being an essential task, the problem of UAV attitude estimation on the basis of sensor measurements can be non-trivial [21], and sensor fault detection must be considered to minimize risks due to loss of control.

In aerospace and several industrial applications, hardware redundancy-based techniques are the most common solutions to tackle faults on sensors. Such solutions require a set of redundant sensors to validate measurement data [22]. A typical approach in commercial and military aircraft is based on triple or more hardware redundancies to enhance safety and reliability [23]. Although hardware redundancy techniques are convenient solutions from the point of view of implementation and management, they bring several drawbacks such as weight, power consumption and costs, which are all relevant in small-size UAV applications.

In the literature, some effort has been spent trying to replace hardware redundancy-based techniques with analytical redundancy-based ones [23,24,25,26,27,28,29,30,31,32]. In [25] a Kalman filtering approach is implemented in order to reveal oscillatory failures of redundant aircraft sensors, used for flight control law calculation. An FDI method that exploits set-valued observers is proposed in [26] for uncertain linear parameter-varying systems. This method uses a bank of filters and it does not need thresholds to state a fault, in contrast with residual-based architectures. A fault-tolerant scheme for the sensors of a large civil aircraft is proposed in [28,29] using Kalman filters and sliding mode observers. Further nonlinear and robust techniques, such as $H_{\infty }$ filtering, can reinforce robustness to faults of physically redundant schemes [32,33].

In recent years, some FDI solutions based on particle filters (PFs) are also proposed. A PF is a sequential Monte Carlo method (SMC)[34] aimed at estimating the state of a dynamical system subject to random perturbations, via noisy output observations. A particle filter solution represents an attractive choice for UAV attitude estimation problems [20,35,36,37,38,39] outperforming classical Kalman filter (KF)-based approaches [40] at the price of significant computational complexity growth [20]. A PF-based fault detection scheme is proposed by [41], while [42] highlights PF advantages in tackling FDI for non-linear systems and [43] also discusses PF drawbacks. In [44] a particle filter deals with the FDI problem for the autonomous integrity monitoring of a GPS receiver. Ref. [45] proposes two different PF-based FDI architectures, which are compared with more classical KF-based approaches. In [46] a particle filter solution is used to deal with both known and unknown faults for a complex system, focusing on a wheeled mobile robot, and in [47] a PF replaces principal component analysis in driving a Gaussian Mixture Model for process monitoring FDI. In [48] a general PF-based FDI scheme is proposed. Similarly [49] is focused upon tackling some of the most well-known PF problems.

In commercial unmanned aviation, costs are a key objective to ensure that UAVs become widespread and, in specific operations, risk assessment needs proof that the flight risk can be acceptable.

In such direction, this paper is focused on the implementation of a sensor fault detection and isolation technique on low-cost hardware, typical of commercial micro- and mini-UAVs. The scope of the proposed procedure is to detect and isolate faults on inertial measurement units used to estimate UAV attitude. The considered hardware is based on a duplex architecture with two inertial measurement units (IMUs) including 3-axis gyroscopes, accelerometers and magnetometers. The estimation problem is dealt with by the design of a particle-filter-based sensor fusion algorithm. From a theoretical point of view, a PF could be a realistic option when statistical performance is considered [50,51,52], because it is able to deal with non-linear motion models and non-Gaussian noise distributions.

Although the use of a PF approach [20] can increase the computational burden, the paper shows promising results by using an SFDI procedure based on two parallel PFs.

The effectiveness of the proposed procedure to detect and isolate IMU sensor faults is proved by means of numerical simulations conducted on a Raspberry Pi board using real data gathered during the flight of a tri-rotor aircraft. Furthermore, this work proposes a sensitivity analysis involving main algorithm parameter variations to estimate effects in terms of SFDI performance. The computation time on a low-cost and low-power embedded platform is assessed in order to prove the algorithm’s applicability in a real-world scenario.

The paper is organized as follows:

- in Section 2, the proposed hardware and software architecture are described;
- in Section 3, the attitude estimation model is defined, using quaternion-based relations;
- in Section 4, the particle filter algorithm is introduced;
- in Section 5, the SFDI algorithm is shown with details on the implementation;
- the effectiveness of the algorithm is shown by means of realistic simulations in Section 6, where an analysis against tunable parameters is discussed.

## 2. System Description

The proposed hardware architecture is composed of the following components:

- two commercially available boards based on the STM32F103VCT CPU by ST, typically used on low-cost multi-rotors as flight controllers; such boards, called IMU-1 and IMU-2, weighing 17 grams, have a power consumption average below 1W and include the following inertial sensors:
  - an MPU6050-based MEMS triaxial accelerometer and gyroscope (see specifications in Table 1);
  - an HMC5883L-based MEMS magnetometer (see specifications in Table 1);
- a Raspberry Pi 3B platform, a single-board computer based on the Broadcom BCM2837 64bit CPU, with wireless LAN, USB and GPIO connectivity. It weighs 42 grams and in our test had a power consumption of less than 2.5W.

IMU-1 and IMU-2 are linked to the Raspberry Pi platform by USB ports through an on-board *serial to USB* chip. This configuration simplified the prototyping while ensuring a high bandwidth for data transmission.

**Figure 1:**
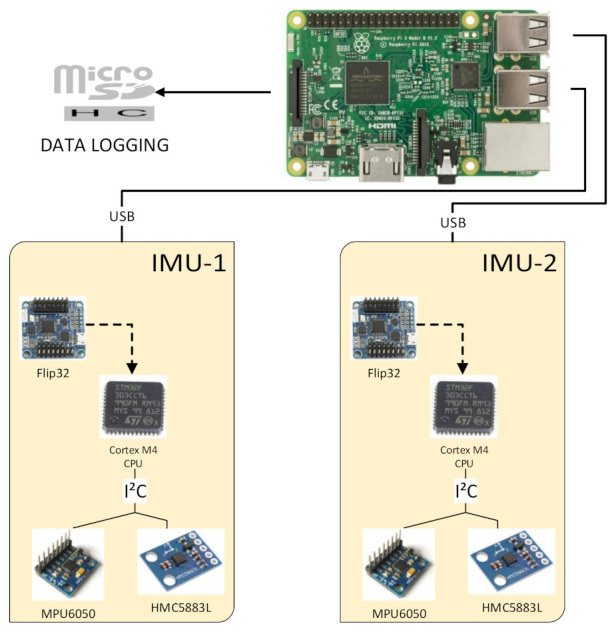
Architecture of the proposed duplex IMU. On top, the Raspberry Pi 3B platform, running the SFDI algorithm and data logging. Below, Flip32-based IMU boards, with MPU6050 and HMC5883L MEMS sensors.

Figure 1 summarizes the structure of the experimental setup.

IMUs act as data collectors. The on-board software acquires signals from sensors on the I${}^{2}$C port at a frequency of 100 Hz and sends them through a serial connection.

The SFDI algorithm, together with particle filtering, was implemented on the Raspberry Pi 3B platform as a real-time task over a Debian-based operating system with a fully preemptive GNU/Linux 4.14 kernel. It was coded using MATLAB R2019b by Mathworks and was made executable on the embedded hardware by using code generation. The produced C code was properly modified to customize peripherals access and recompiled on the board. During our tests, the SFDI executable had the highest execution priority to minimize any jitter due to the operating system.

The goal of the SFDI algorithm is to monitor the health of each IMU and to supervise the overall system by providing reliable data to the flight control system. In standard conditions, the output of the SFDI algorithm is the average attitude estimated using both IMUs, while, when a fault is detected, the procedure provides as outputs only the orientation computed by the operational IMU.

## 3. UAV Attitude Estimation Problem

Vehicle attitude is defined as its orientation with respect to a reference frame and its accurate estimation is an important topic in the robotics and aerospace fields. UAV attitude is obtained by using a sensor fusion algorithm, together with data provided by IMU sensors.

The attitude obtained by integrating gyro measurements in terms of angular velocities is affected by non-negligible issues, due to bias and random-walk errors. On the other hand, accelerometers and magnetometers provide a good estimate only in static or quasi-static conditions, in the absence of any constant acceleration and magnetic field changes. In order to mitigate such problems, different sensor data fusion algorithms can be implemented on-board [53,54]. Most UAV attitude estimation algorithms are based on an extended Kalman filter [20,55,56,57], unscented Kalman filter [58,59], particle filtering [36,51,60], quaternion estimation (QUEST) algorithms [61,62], complementary filter [63,64] and Madgwick filter [65].

To describe the attitude estimation problem, two reference systems must be defined:

- a north-east-down (NED) reference frame, parallel to the Earth’s surface, with the $X_{E}$-axis pointed toward north, the $Y_{E}$-axis pointed toward east and the $Z_{E}$-axis oriented downwards;
- the body frame, centered in the UAV’s center of gravity (CG), with the $X_{B}$-axis pointed toward the UAV nose, the $Z_{B}$-axis downward and the $Y_{B}$-axis oriented to complete a right-handed system.

Since UAV navigation is done in a bounded space, the NED frame can be centered in the UAV departing point and assumed as an inertial reference system.

**Figure 2:**
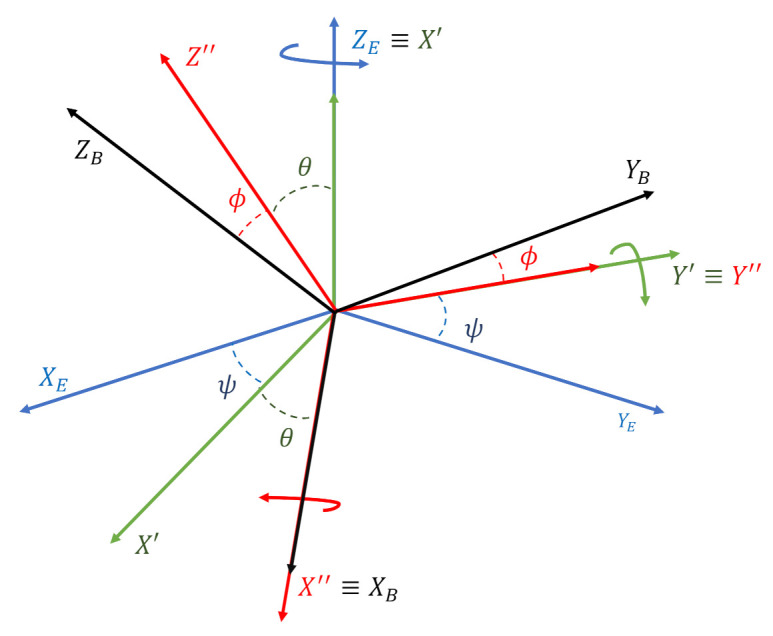
Inertial (E) and body-fixed (B) reference frames. Transition from the NED frame to the body frame is obtained with a sequence of three ordered rotations around the $Z_{E}$ axis, the $Y^{\prime }$ axis and the $X^{\prime \prime }$ axis of angles ψ, θ and ϕ, respectively.

Transition from the inertial to the body frame is obtained with a sequence of three ordered rotations, denoted as 3−2−1 [66] (see Figure 2):

- the first rotation around the $Z_{E}$ axis by the yaw angle ψ, from $OX_{E}Y_{E}Z_{E}$ to $OX^{\prime }Y^{\prime }Z^{\prime }$;
- the second rotation around the $Y^{\prime }$ axis by the pitch angle θ from $OX^{\prime }Y^{\prime }Z^{\prime }$ to $OX^{\prime \prime }Y^{\prime \prime }Z^{\prime \prime }$;
- the third rotation around the $X^{\prime \prime }$ axis by the roll angle ϕ from $OX^{\prime \prime }Y^{\prime \prime }Z^{\prime \prime }$ to $OX_{B}Y_{B}Z_{B}$.

The angles ψ, θ and ϕ are called Euler angles and describe the aircraft’s attitude, i.e., the orientation of the body frame with respect to the inertial NED frame.

**Figure 3:**
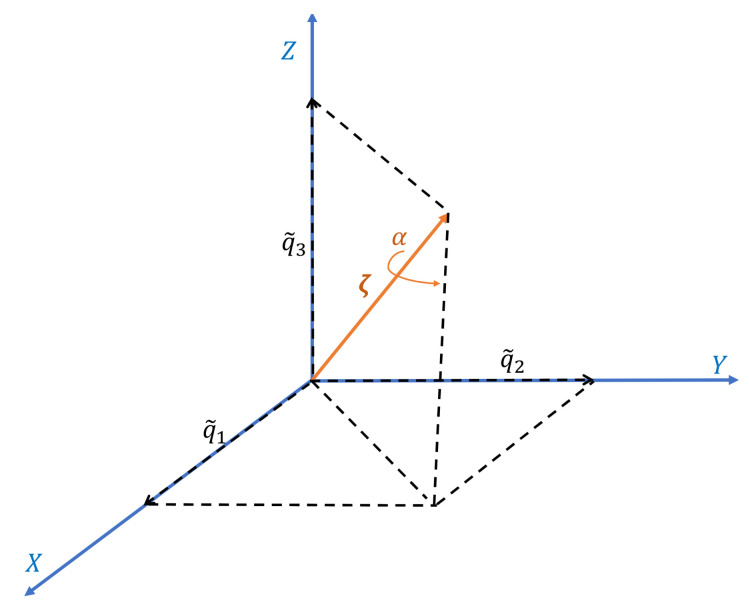
Geometrical meaning of quaternion: a rotation around the ζ axis by a given angle α is defined by the unit quaternion $\tilde{q}={\left[{\tilde{q}}_{0},{\tilde{q}}_{1},{\tilde{q}}_{2},{\tilde{q}}_{3}\right]}^{T}$, whose components are $\tilde{q_{1}}=\zeta_{x}$, $\tilde{q_{2}}=\zeta_{y}$, $\tilde{q_{3}}=\zeta_{z}$ and $\tilde{q_{0}}=\cos\alpha$.

Alternatively, to define the relative rotation of the body frame with respect to the inertial frame, a quaternion-based representation can be used [67]. From Euler’s theorem, any rotation sequence is equivalent to a single rotation by a given angle α about a fixed axis passing through the origin. This rotation can be described by the unit quaternion vector $\tilde{q}={\left[\tilde{q_{0}},\tilde{q_{1}},\tilde{q_{2}},\tilde{q_{3}}\right]}^{T}$, whose components define the axis vector $\zeta ={\left[\zeta_{x},\zeta_{y},\zeta_{z}\right]}^{T}$ and the angle α (see Figure 3):

$$\begin{array}{cc} \tilde{q_{1}} & =\zeta_{x},\;\tilde{q_{2}}=\zeta_{y},\;\tilde{q_{3}}=\zeta_{z},\;\tilde{q_{0}}=\cos\alpha \\ & |\tilde{q}|=\sqrt{{\tilde{q_{0}}}^{2}+{\tilde{q_{1}}}^{2}+{\tilde{q_{2}}}^{2}+{\tilde{q_{3}}}^{2}} \end{array}$$

Let us call $q={\left[q_{0},q_{1},q_{2},q_{3}\right]}^{T}$ the unit quaternion vector associated to $R_{\mathrm{BE}}\left(q\right)$ from the NED reference frame to the body frame:

(1) $$R_{\mathrm{BE}}\left(q\right)=\left[\begin{array}{ccc} q_{1}^{2}-q_{2}^{2}-q_{3}^{2}+q_{0}^{2} & 2(q_{1}q_{2}-q_{3}q_{0}) & 2(q_{1}q_{3}+q_{2}q_{0})\\ 2(q_{1}q_{2}+q_{3}q_{0}) & -q_{1}^{2}+q_{2}^{2}-q_{3}^{2}-q_{0}^{2} & 2(q_{2}q_{3}-q_{1}q_{0})\\ 2(q_{1}q_{3}-q_{2}q_{0}) & 2(q_{2}q_{3}+q_{1}q_{0}) & -q_{1}^{2}-q_{2}^{2}+q_{3}^{2}+q_{0}^{2} \end{array}\right]$$

Euler angle-based representation and quaternion-based representation are equivalent; therefore, a one-to-one correspondence between Euler angles and the quaternion vector exists:

(2) $$q=\left[\begin{array}{c} q_{0}\\ q_{1}\\ q_{2}\\ q_{3} \end{array}\right]=\left[\begin{array}{c} \cos\frac{\phi }{2}\cos\frac{\theta }{2}\cos\frac{\psi }{2}+\sin\frac{\phi }{2}\sin\frac{\theta }{2}\sin\frac{\psi }{2}\\ \sin\frac{\phi }{2}\cos\frac{\theta }{2}\cos\frac{\psi }{2}-\cos\frac{\phi }{2}\sin\frac{\theta }{2}\sin\frac{\psi }{2}\\ \cos\frac{\phi }{2}\sin\frac{\theta }{2}\cos\frac{\psi }{2}-\sin\frac{\phi }{2}\cos\frac{\theta }{2}\sin\frac{\psi }{2}\\ \cos\frac{\phi }{2}\cos\frac{\theta }{2}\sin\frac{\psi }{2}-\sin\frac{\phi }{2}\sin\frac{\theta }{2}\cos\frac{\psi }{2} \end{array}\right]$$

The Euler angles are computed as follows [66]:

(3) $$\begin{array}{ccc} \phi & = & \arctan\left(\frac{2q_{0}q_{1}+2q_{2}q_{3}}{1-2q_{1}^{2}-2q_{2}^{2}}\right) \end{array}$$

(4) $$\begin{array}{ccc} \theta & = & \arcsin\left(2q_{0}q_{2}-2q_{3}q_{1}\right) \end{array}$$

(5) $$\begin{array}{ccc} \psi & = & \arctan\left(\frac{2q_{0}q_{3}+2q_{1}q_{2})}{1-2q_{2}^{2}-2q_{3}^{2}}\right) \end{array}$$

In the absence of noise and uncertainties in IMU measurements, the UAV attitude estimation could be carried out by integrating the angular speed vector $\omega ={\left[p,q,r\right]}^{T}$ from a known initial attitude. Consequently, the quaternion vector exhibits the following dynamics:

(6) $$\dot{q}\left(t\right)=\frac{1}{2}Q\left(\omega \left(t\right)\right)\cdot q\left(t\right)$$

where the matrix Q(ω(t)) is

(7) $$Q\left(\omega \left(t\right)\right)=\left[\begin{array}{cccc} 0 & -p(t) & -q(t) & -r(t)\\ p(t) & 0 & r(t) & -q(t)\\ q(t) & -r(t) & 0 & p(t)\\ r(t) & q(t) & -p(t) & 0 \end{array}\right]$$

However, the gyroscope output is an angular velocity affected by white noise. In static conditions, in the absence of rotations, the gyroscope output is not zero as expected, but it is a white noise with a zero mean and a given standard deviation. On a finite time horizon, the integration will lead to a drifting of the angle estimation [68,69].

The sensed rotational velocities $\omega_{S}\left(t\right)$ can be assumed different from ω due to the presence of bias and stochastic noise:

(8) $$\omega_{s}\left(t\right)=\left[\begin{array}{c} p_{s}\left(t\right)\\ q_{s}\left(t\right)\\ r_{s}\left(t\right) \end{array}\right]=\omega \left(t\right)+b\left(t\right)+d\left(t\right)$$

$b\left(t\right)={\left[b_{p}\left(t\right),b_{q}\left(t\right),b_{r}\left(t\right)\right]}^{T}$ and $d\left(t\right)={\left[d_{p}\left(t\right),d_{q}\left(t\right),d_{r}\left(t\right)\right]}^{T}$ represent the vectors of biases and unknown zero-mean noise affecting gyroscopes, respectively.

In steady-state and slowly varying conditions, a good solution is to involve accelerometers and magnetometers, being dependent on the UAV orientation. The relationship between UAV orientation and accelerometer and magnetometer output vectors $a^{B}$ and $M^{B}$ is defined as follows:

(9) $$a^{B}=R_{\mathrm{BE}}\left(q\right)\cdot a^{E}$$

(10) $$M^{B}=R_{\mathrm{BE}}\left(q\right)\cdot M^{E}$$

where $g^{E}={\left[\begin{array}{ccc} 0 & 0 & g \end{array}\right]}^{T}$ is the gravity vector with g=9.801 m/s${}^{2}$ and $M^{E}$ is the Earth’s magnetic field, both defined in the Earth frame.

However, although an optimization-based procedure can be used to solve Equations (9) and (10), the last approach is not reliable [67,70], due to the presence of non-negligible noise in measurements.

In order to exploit (9) and (10), consider the sensed accelerations and field magnetic measurements:

(11) $$a_{S}\left(t\right)=\left[\begin{array}{c} a_{x,S}\left(t\right)\\ a_{y,S}\left(t\right)\\ a_{z,S}\left(t\right) \end{array}\right]=a^{B}\left(t\right)+v_{a}\left(t\right)$$

(12) $$M_{S}\left(t\right)=\left[\begin{array}{c} M_{x,S}\left(t\right)\\ M_{y,S}\left(t\right)\\ M_{z,S}\left(t\right) \end{array}\right]=M^{B}\left(t\right)+v_{M}\left(t\right)$$

with $v_{a}\left(t\right)$ and $v_{M}\left(t\right)$ vectors of sensor noise affecting triaxial accelerometers and magnetometers at the time instant *t*, respectively.

The attitude estimation can be carried out by merging both approaches [20,71]. The kinematic model (6) is usually extended with additional dynamics in order to take into account the gyroscope biases [72]:

(13) $$\dot{q}\left(t\right)=\frac{1}{2}Q\left(\omega_{S}\left(t\right)-b(t)-d(t)\right)\cdot q\left(t\right)$$

(14) $$\dot{b}\left(t\right)=-\frac{1}{\tau }b\left(t\right)$$

where τ is a time constant compliant with bias dynamics. To correct the previous estimation, (15) and (16) can be used:

(15) $$a_{S}\left(t\right)=R_{BE}\left(q\left(t\right)\right)\cdot g^{E}+v_{a}\left(t\right)$$

(16) $$M_{S}\left(t\right)=R_{BE}\left(q\left(t\right)\right)\cdot M^{E}+v_{M}\left(t\right)$$

Equations (13)–(16) can be expressed in the following generic form:

(17) $$\dot{x}\left(t\right)=\gamma \left(x(t),\omega_{S}\left(t\right),d(t)\right)$$

(18) y(t)=λ(x(t),v(t))

where $x\left(t\right)={\left[\begin{array}{cc} q\left(t\right){\;}^{T} & b\left(t\right){\;}^{T} \end{array}\right]}^{T}$ is the state vector, $y\left(t\right)={\left[\begin{array}{cc} a_{S}\left(t\right){\;}^{T} & M_{S}\left(t\right){\;}^{T} \end{array}\right]}^{T}$ is the output vector and $v\left(t\right)={\left[\begin{array}{cc} v_{a}{\left(t\right)}^{T} & v_{M}{\left(t\right)}^{T} \end{array}\right]}^{T}$ is the noise vector.

In such representation, accelerometers and magnetometers provide output sensor measurements according to (18), whereas gyroscopes represent an external input acting on (17).

The attitude estimation problem can be reformulated as the estimation of the state vector x from (17) based on output measurements (18). Corrective actions can then be designed and implemented, feeding back the error between measured and estimated outputs. Optimal estimation has the role of reducing sensitivity to noise and forcing the additional state vector components b to compensate for gyro biases or other low-frequency uncertain contributions.

## 4. Particle Filter Algorithm

Let us assume the following generic discrete time representation:

(19) $$x\left(k\right)=f(x\left(k-1\right),\omega_{S}\left(k-1\right),d\left(k-1\right))$$

(20) y(k)=h(x(k))+v(k)

being $x\in R^{n_{x}}$, $y\in R^{n_{y}}$, $\omega_{s}\in R^{n_{\omega }}$, $v\in R^{n_{v}}$ and $d\in R^{n_{d}}$. It can be obtained by applying Euler’s discretization to (17) and (18) where $x\left(k\right)=x\left(t_{k}\right)$, $y\left(k\right)=y\left(t_{k}\right)$, $\omega_{S}\left(k\right)=\omega_{S}\left(t_{k}\right)$, $d\left(k\right)=d\left(t_{k}\right)$ and $v\left(k\right)=v\left(t_{k}\right)$ and $t_{k}$ the k−th sampling time instant

(21) $$t_{k}=\sum_{i=0}^{k-1}T_{S}\;\;\;\forall k\geq 1,\;\;\;t_{0}=0$$

with $T_{S}>0$ the constant sampling time.

Equations (19) and (20) represent a partially observed Markov process, where the output vector y(·) is observable, while the state x(·) is unobservable. A Markov process is a stochastic process whose state vector x(k) depends only on x(k−1), being independent of x(k−l), with l>1.

A particle filter is used to estimate x(k) in (19), fulfilling (20) under the hypothesis that the process d(k−1) and the measurement noise v(k) are unknown.

The particle filter is a numeric implementation of a Bayesian estimator [73]. The Bayesian approaches are based on Bayes’ rule. Their goal is to estimate the conditional probability density function (PDF) of the current state x(k), given the whole set of acquired measurements at time *k*, denoted by Y(k)={y(0),y(1),⋯,y(k)}. The conditional PDF is denoted as $p\left(x(k)|Y(k)\right)$.

The Bayesian estimator can be formulated in a recursive way, in order to update the PDF when a new measurement is acquired [74].

At the first time step k=0, since no measurements are available, e.g., the set Y(0)=∅, the estimator is initialized as follows:

(22) $$p\left((x)(0)|Y(0)\right)=p\left(x(0)\right)$$

where $p\left(x(0)\right)$ is the known initial PDF of state.

At each time step *k*, the a priori PDF denoted by $p\left(x(k)|Y(k-1)\right)$ is computed using the Chapman–Kolmogorov formula [73]:

(23) $$p\left(x(k)|Y(k-1)\right)=\int p\left(x(k)|x(k-1)\right)\cdot p\left(x(k-1)|Y(k-1)\right)\cdot dx\left(k-1\right)$$

In the a posteriori phase, the conditional PDF is computed by using Bayes’ rule as follows:

(24) $$p\left(x(k)|Y(k)\right)=\frac{p\left(y(k)|x(k)\right)\cdot p\left(x(k)|Y(k-1)\right)}{p\left(y(k)|Y(k-1)\right)}$$

where $p\left(y(k)|x(k)\right)$ is available thanks to measurement equations and the PDF of the measurement noise v(k).

The PDF $p\left(y(k)|Y(k-1)\right)$ depends on the a priori PDF $p\left(x(k)|Y(k-1)\right)$ and $p\left(y(k)|x(k)\right)$:

(25) $$p\left(y(k)|Y(k-1)\right)=\int p\left(x(k)|x(k-1)\right)\cdot p\left(x(k-1)|Y(k-1)\right)\cdot dx\left(k-1\right)$$

Equations (23)–(25) involve intractable multidimensional integrals [34]. The particle filtering approach offers a numerical solution to this problem [75].

In PF algorithms, PDF p(x(k)|y(k)) can be approximated by a set of random samples named *particles*. According to [76], the convergence rate of the particle-approximated cumulative function is $O\left(\frac{1}{\sqrt{\Delta }}\right)$, with Δ a constant tunable parameter indicating the number of particles.

An important feature that characterizes particle filter algorithms is the method used to obtain new samples. A widely used class of resampling techniques is *sequence importance resampling* (SIR) [77].

Let us assume discrete-time model (19) and (20). A particle filter is used to estimate the x(k) value knowing the vector y(k) of accelerometer and magnetometer measurements and the vector $\omega_{S}\left(k-1\right)$ of the rotational speed sensed by the gyroscopes.

Assuming that the PDF of the initial state p(x(0)) is known, the PF algorithm is initialized randomly generating Δ particles $\hat{x}\left(0\right)$, on the basis of PDF p(x(0)). Then, at each time step *k*, the procedure involves the following steps:

(i) the set of a priori particles X(k|k−1) at the *k*-th sampling step is obtained by propagating the set X(k−1|k−1) with the state Equation (19), considering the process noise d(k) equal to zero. Therefore, the generic particle $x_{\delta }\left(k\right)\in X\left(k|k-1\right)$ is computed as follows:
  (26) $$\begin{array}{c} x_{\delta }\left(k\right)=f\left({\hat{x}}_{\delta }\left(k-1\right),\omega_{S}\left(k-1\right),0\right) \end{array}$$
  being ${\hat{x}}_{\delta }\left(k-1\right)\in X\left(k-1|k-1\right)$ with δ=1,⋯,Δ.
(ii) We compute the relative likelihood ${\tilde{w}}_{\delta }\left(k\right)$ of each particle $x_{\delta }\left(k\right)$ conditioned on the measurement y(k)., i.e., evaluate the PDF $p\left(y\left(k\right)|x_{\delta }\left(k\right)\right)$ on the basis of the nonlinear measurement equation and the PDF of the measurement noise v(k). Under the assumption of additive measurement noise, the weight ${\tilde{w}}_{\delta }\left(k\right)$ can be computed as follows:
  (27) $${\tilde{w}}_{\delta }\left(k\right)=P\left[y\left(k\right)|x_{\delta }\left(k\right))\right]=P\left[v\left(k\right)=\left(y\left(k\right)-y_{\delta }\left(k\right)\right)\right]$$
  where P(·) denotes the probability and $y_{\delta }$ is obtained according to the output equation, neglecting the measurement noise v(k):
  (28) $$y_{\delta }\left(k\right)=h\left(x_{\delta }\left(k\right)\right)$$
(iii) The weights ${\tilde{w}}_{\delta }\left(k\right)$ are normalized according to the following equation:
  (29) $$w_{\delta }\left(k\right)=\frac{{\tilde{w}}_{\delta }\left(k\right)}{\sum_{\delta =1}^{\Delta }{\tilde{w}}_{\delta }\left(k\right)}$$
(iv) A set of particles taking into account a posteriori knowledge $\tilde{X}\left(k|k\right)$ is obtained by randomly drawing Δ samples from X(k|k−1)
  (30) $$\begin{array}{cc} \tilde{X}\left(k|k\right)=\left\{x_{\delta }\left(k\right)\in X\left(k|k-1\right),\underset{m_{1}}{\underset{︸}{x_{1}\left(k\right),\cdots ,x_{1}\left(k\right)}},\cdots ,\right. & \\ \left.\underset{m_{\delta }}{\underset{︸}{x_{\delta }\left(k\right),\cdots ,x_{\delta }\left(k\right)}},\cdots ,\underset{m_{\Delta }}{\underset{︸}{x_{\Delta }\left(k\right),\cdots ,x_{\Delta }\left(k\right)}}\right\} \end{array}$$
  therefore, each particle $x_{\delta }\in X\left(k|k-1\right)$ is chosen $m_{\delta }$ times. The integer number $m_{\delta }$ must satisfy the following conditions:
  - $m_{\delta }$ is a non-negative integer;
  - $\sum_{\delta =1}^{\Delta }m_{\delta }=\Delta$;
  - $\lim_{\Delta \to \infty }m_{\delta }=w_{\delta }\left(k\right)\cdot \Delta$.
  To compute the parameter $m_{\delta }$ the following sub-procedure is applied:
  (a) define a series of Δ+1 thresholds $\sigma_{\delta }$ as:
    (31) $$\sigma_{\delta }=\sum_{1}^{\delta }w_{\delta },\;\mathrm{with}\;\sigma_{0}=0$$
  (b) initialize $m_{\delta }$ values to zero;
  (c) draw Δ uniformly distributed random numbers $r_{i}$ from the interval [0,1], $P=\{r_{i}\in \left[0,1\right],\mathrm{with}\;i=1,...,\Delta \}$;
  (d) for each $r_{i}\in P$, find δ such that $\sigma_{\delta -1}<r_{i}\leq \sigma_{\delta }$, then $m_{\delta }=m_{\delta }+1$.
(v) In order to counteract the so-called *particle degeneracy problem*, which can weaken algorithm convergence and robustness [78], the set of a posteriori particle X(k|k) is obtained by randomly scattering each particle of set $\tilde{X}\left(k|k\right)$ in a given neighborhood.
(vi) The state estimate $\hat{x}\left(k\right)$ is evaluated as the algebraic mean of a posteriori particles belonging to X(k|k):
  (32) $$\hat{x}\left(k\right)=\frac{1}{\Delta }\sum_{\delta =1}^{\Delta }{\hat{x}}_{\delta }\left(k\right)$$

In Section 6.2 a sensitivity analysis against introduced tunable parameters Δ and ν is proposed.

## 5. SFDI Algorithm for a Duplex IMU

In the view of accomplishing navigation purposes, let us consider an onboard flight controller based on a *duplex sensor architecture* including two IMUs, named IMU-1 and IMU-2, with triaxial accelerometers, gyroscopes and magnetometers, whose output sensor measurements $y^{\left(1\right)}\left(k\right)$ and $y^{\left(2\right)}\left(k\right)$ are defined as follows:

(33) $$y^{\left(i\right)}\left(k\right)=\left[\begin{array}{c} a_{S}^{\left(i\right)}\left(k\right)\\ M_{S}^{\left(i\right)}\left(k\right) \end{array}\right]\;i=1,2$$

where $a_{S}^{\left(i\right)}\left(k\right)$, $M_{S}^{\left(i\right)}\left(k\right)$ are the outputs of the *i*-th triaxial accelerometer and magnetometer at the time instant *k*.

According to (8), the gyroscope measurements $\omega_{S}^{\left(i\right)}\left(k\right)$ are used as the input of the kinematic model at the time instant *k*.

For each IMU *i*, the quaternion vector $q^{\left(i\right)}\left(k\right)$ and gyroscope biases $b^{\left(i\right)}\left(k\right)$ are estimated by using a particle filter, named *PFi*, fed with $\omega_{S}^{\left(i\right)}\left(k\right)$ and $y^{\left(i\right)}\left(k\right)$.

A sensor reconfiguration logic has been implemented to achieve FDIR, formulated as an *event-driven* state machine, based on the following sets of logical states:

(34) $$\chi \left(k\right)=\left[N_{A}\left(k\right),A_{A}\left(k\right),N_{G}\left(k\right),A_{G}\left(k\right),N_{M}\left(k\right),A_{M}\left(k\right),F_{1}\left(k\right),F_{2}\left(k\right),R_{1}\left(k\right),R_{2}\left(k\right)\right]$$

where

- subscript *A* indicates accelerometers, subscript *G* is for gyroscopes and subscript *M* is for magnetometers;
- $N_{Z}$ is the normal state for sensor Z∈{A,G,M};
- $A_{Z}$ is the alert state activated at the detection phase on the basis of a preliminary comparison between IMUs;
- $F_{j}$ is the fault state for the IMU unit j∈{1,2}.

**Figure 4:**
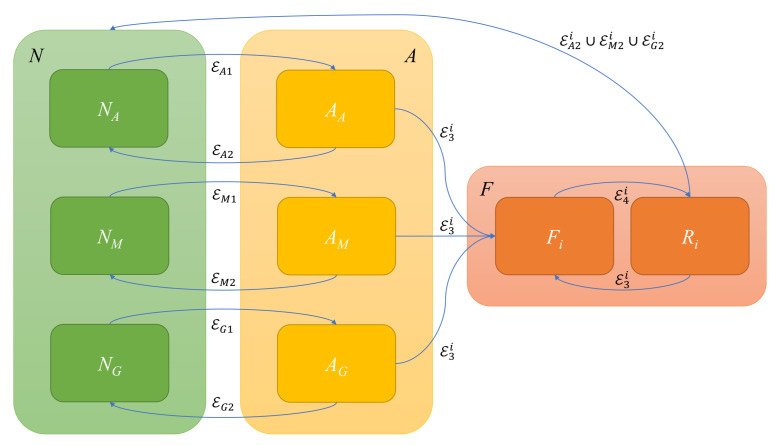
State transition graph for SFDI. Normal state (green) $N=N_{A}\cap N_{M}\cap N_{G}$, alert state (yellow) $A=A_{A}\cap A_{M}\cap A_{G}$, fault state (red) $F=\left({⋂}_{i=1}^{2}F_{i}\right)\cap \left({⋂}_{i=1}^{2}R_{i}\right)$. Arrows denote event-driven state transitions.

In Figure 4, the state transition graph is shown. The events driving the state transition of the proposed state machine can be grouped into three algorithm steps:

**Step 1—FAULT DETECTION.** Under the assumption of a non-contemporary fault, at each time step *k*, the transition from normal N(k−1) to alert A(k) state (and vice versa from A(k−1) to N(k)) is achieved by comparing measurement accelerometer, magnetometer and gyroscope measurements from IMUs.

(35) $$\begin{array}{ccc} E_{A1} & \Leftrightarrow & \exists l\in \left\{1,2,3\right\}:\;|a_{l,S}^{\left(1\right)}\left(k\right)-a_{l,S}^{\left(2\right)}\left(k\right)|\geq \tau_{a}\;\;\; \end{array}$$

(36) $$\begin{array}{ccc} E_{A2} & \Leftrightarrow & \forall l\in \left\{1,2,3\right\},\;|a_{l,S}^{\left(1\right)}\left(k\right)-a_{l,S}^{\left(2\right)}\left(k\right)|<\tau_{a}\;\;\; \end{array}$$

(37) $$\begin{array}{ccc} E_{M1} & \Leftrightarrow & \exists l\in \left\{1,2,3\right\}:|M_{l,S}^{\left(1\right)}\left(k\right)-M_{l,S}^{\left(2\right)}\left(k\right)|\geq \tau_{m}\;\;\; \end{array}$$

(38) $$\begin{array}{ccc} E_{M2} & \Leftrightarrow & \forall l\in \left\{1,2,3\right\},|M_{l,S}^{\left(1\right)}\left(k\right)-M_{l,S}^{\left(2\right)}\left(k\right)|<\tau_{m}\;\;\; \end{array}$$

(39) $$\begin{array}{ccc} E_{G1} & \Leftrightarrow & \exists l\in \left\{1,2,3\right\}:|\omega_{l,S}^{\left(1\right)}\left(k\right)-\omega_{l,S}^{\left(2\right)}\left(k\right)|\geq \tau_{g}\;\;\; \end{array}$$

(40) $$\begin{array}{ccc} E_{G2} & \Leftrightarrow & \forall l\in \left\{1,2,3\right\},|\omega_{l,S}^{\left(1\right)}\left(k\right)-\omega_{l,S}^{\left(2\right)}\left(k\right)|<\tau_{g}\;\;\; \end{array}$$

where $a_{l,s}$, $M_{l,s}$ and $\omega_{l,s}$ are the *l*-th components of accelerometer, magnetometer and gyroscope measurements, respectively. $\tau_{a}$, $\tau_{m}$ and $\tau_{g}$ are positive scalar thresholds for accelerometers, magnetometers and gyroscopes, required to make the detection robust against sensor noise and uncertainties.

**Step 2—FAULT ISOLATION.** Once the presence of a fault is detected, with $\tilde{k}$ the time instant of fault detection, then

$$b_{0}^{\left(i\right)}=b^{\left(i\right)}\left(\tilde{k}\right)$$

As for the transition from the alert to the faulted state, a fault on the *i*-th IMU is declared if the following condition holds:

(41) $$E_{3}^{i}\Leftrightarrow ||\Omega \cdot \left(q^{\left(1\right)}\left(k\right)-q^{\left(2\right)}\left(k\right)\right){\left|\right|}_{\infty }>1$$

where $\left|\right|\cdot {\left|\right|}_{\infty }$ denotes the infinity norm and Ω is a diagonal weighing matrix required to take into account the effects of sensor noise. The faulted IMU $\bar{i}$ can be isolated solving the following problem:

(42) $$\bar{i}=\underset{i=1,2}{arg max}\sigma \left(i\right)$$

The residual σ(i) represents the error between output estimation and measurements, increased with the difference between the estimated biases at the current time and at the detection time:

(43) $$\sigma \left(i\right)={\left|\left|\left[\begin{array}{c} y^{\left(i\right)}\left(k\right)\\ b_{0}^{\left(i\right)} \end{array}\right]-\left[\begin{array}{c} {\hat{y}}^{\left(i\right)}\left(k\right)\\ {\hat{b}}^{\left(i\right)}\left(k\right) \end{array}\right]\right|\right|}_{2}\;i=1,2$$

where $\left|\right|\cdot {\left|\right|}_{2}$ indicates the Euclidean norm.

According to (42), a faulted sensor belongs to the IMU whose output is farther from the relevant full particle filter forecast.

**Step 3—FAULT RECOVERY.** When the fault condition is no longer held ($E_{Z2}$), the system is able to recover the faulted IMU by checking the consistency between attitude estimations from both *PFs*, through

(44) $$E_{4}^{\bar{i}}\Leftrightarrow ||\Omega \cdot \left(q^{\left(1\right)}\left(k\right)-q^{\left(2\right)}\left(k\right)\right){\left|\right|}_{\infty }<1$$

In Section 6.2 a sensitivity analysis against introduced tunable parameters $\tau_{a}$, $\tau_{m}$, $\tau_{g}$ and Ω is proposed.

## 6. Numerical Simulations Based on Experimental Data

**Figure 5:**
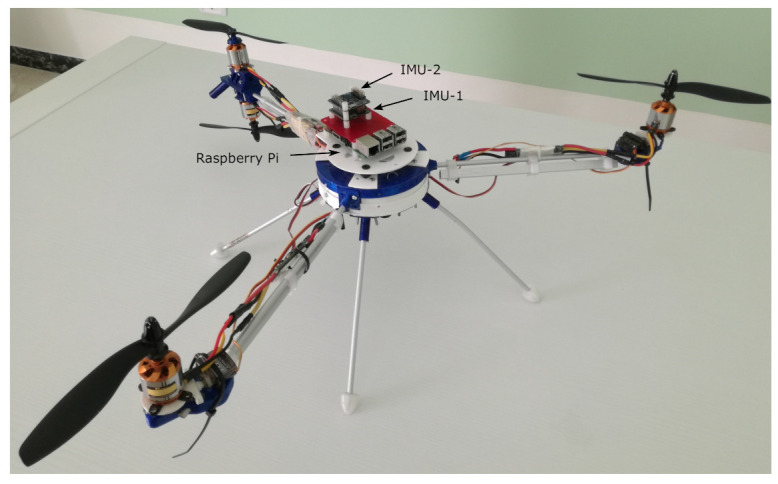
A picture of the custom tri-rotor aircraft used in the flight test with the duplex hardware platform on top.

Experimental validation of the proposed approach was carried out with the aim of proving its effectiveness along with its applicability to a low-cost small-UAV architecture. For this purpose, the hardware platform presented in Section 2 was installed on a light tri-rotor aircraft of less than 2 kg (see Figure 5), to gather in-flight sensor data.

The tri-rotor platform has an on-board flight controller [79], used to perform an indoor flight, forcing the system to follow pre-assigned reference angles and altitudes.

**Figure 6:**
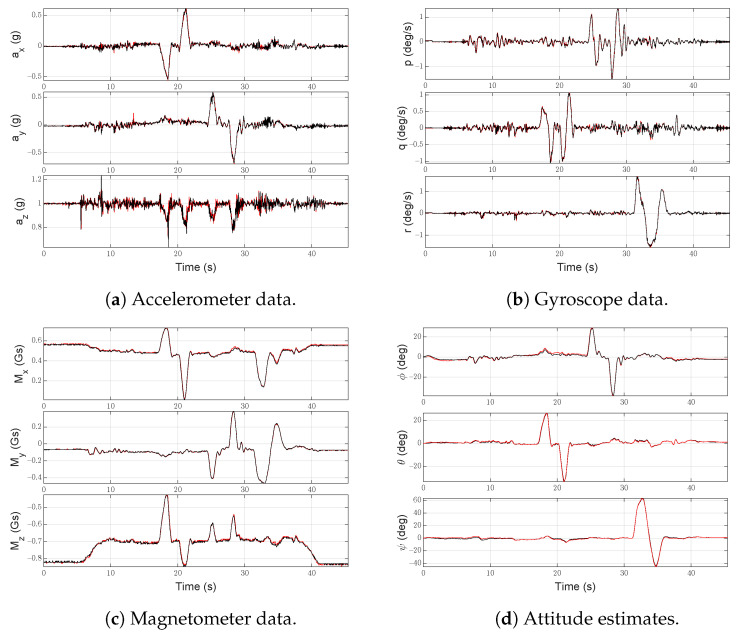
Data gathered from both IMUs during the flight test in the absence of faults. Black lines indicate data gathered by IMU-1 while red lines are for IMU-2.

Captured data from both IMUs is shown in Figure 6. The estimated attitude in the absence of any fault is shown in Figure 6d. Three maneuvers were performed, sequentially changing Euler angles:

- a backward movement, executed by applying a doublet (+/−) to the pitch angle;
- a lateral movement, with a doublet (+/−) applied to the roll angle;
- a doublet applied to the yaw angle.

### 6.1. Fault Scenario Analyses

Experimental data gathered during the flight were used as measurements in numerical simulations, where realistic faults were injected to test the SFDI algorithm.

Typical fault scenarios [80,81,82,83,84] were considered:

- *F1: intermittent abrupt bias*—a step disturbance of 0.2 g is added to $a_{y,S}^{\left(1\right)}\left(t\right)$ for t∈[3,13] s;
- *F2: slow drift*—a linearly increasing signal with a rate of 0.2 rad/s${}^{2}$ is added to the measured yaw rate on IMU-2 starting from the time instant t=3.5 s;
- *F3: abrupt freezing*—magnetometer data $M_{x,S}^{\left(1\right)}\left(t\right)$ stops being updated at t=3 s;
- *F4: oscillation*—a sinusoidal signal with a frequency of 30 rad/s is added to the $r_{S}^{\left(1\right)}\left(t\right)$ measurement for t∈[3,28] s;
- *F5: random walk*—Gaussian noise, whose amplitude is smaller than the relevant detection threshold, is integrated and added to accelerometer data $a_{x,S}\left(t\right)$ for t∈[23,33] s.

Table 2 resumes the values of the SFDI scheme design parameters, as introduced in Section 4 and Section 5.

#### 6.1.1. Scenario F1—Intermittent Abrupt Bias

**Figure 7:**
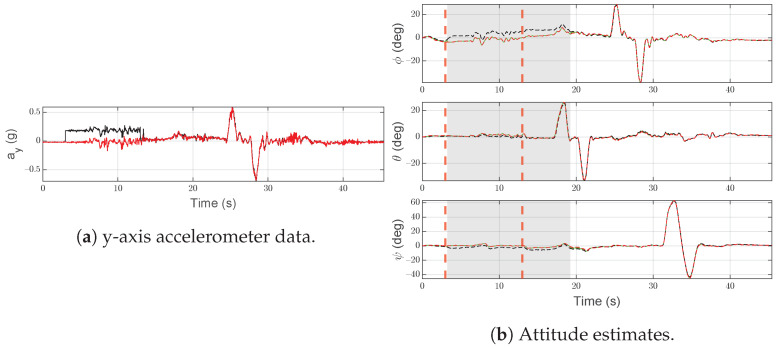
Scenario F1. Black lines denote measurements/estimates from IMU-1, red lines are for measurements/estimates from IMU-2. Green lines are for SFDI output, gray areas highlight the time interval in which the SFDI algorithm is in the fault state. Vertical red dashed lines denote the time instants when the fault occurs and ends.

The intermittent abrupt bias on $a_{y,S}^{\left(1\right)}\left(t\right)$ is depicted in Figure 7a. As shown in Figure 7b, the attitude estimated by IMU-1 is affected by an error that mostly affects the roll angle. In Figure 7b, vertical dashed red lines indicate the range in which the fault is injected and the gray area is used to highlight the time interval in which the SFDI algorithm is in the fault state.

As expected, the considered fault is quickly ($t_{d}=0.27$ s) detected and IMU-2 is correctly labeled as the operational unit (see red line in Figure 7b). Furthermore, at t=13 s, the accelerometer returns to operational and after 6.96 s the SFDI algorithm returns to the normal state, restoring estimated attitude based on IMU-1 measurements. The time required to restore the estimated attitude based on IMU-1 depends on the design parameters, see Section 6.2. However, it does not affect the performance of the estimated attitude, given the presence of IMU-2.

#### 6.1.2. Scenario F2—Slow Drift

**Figure 8:**
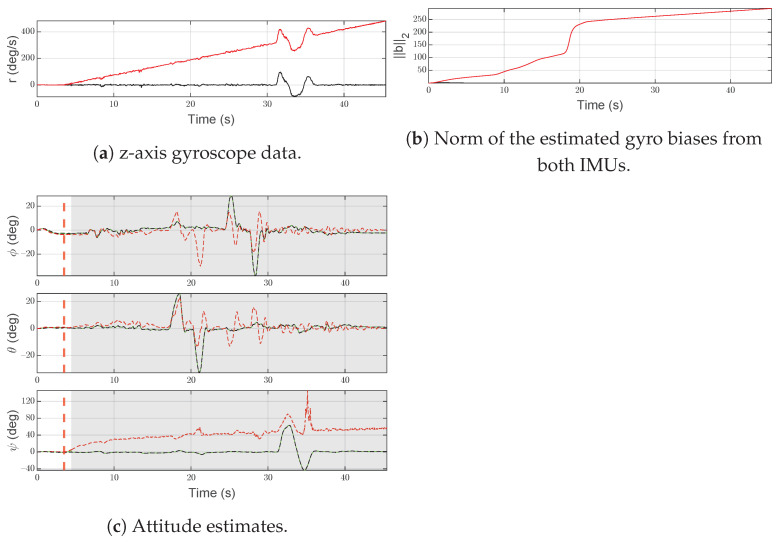
Scenario F2. Black lines denote measurements/estimates from IMU-1, red lines are for measurements/estimates from IMU-2. Green lines represent the output of the system, gray areas highlight the time interval in which the SFDI algorithm is in the fault state. Vertical red dashed lines denote the time instants when the fault occurs and ends.

In Figure 8a, the slow drift injected at time t=3.5 s, with a slope of 0.2 rad/s${}^{2}$, into the *z*-axis gyroscope measurement $r_{S}^{\left(2\right)}\left(t\right)$ of IMU-2 is shown.

As shown in Figure 8c, at time t=4.61 s, the SFDI scheme isolates the gyroscope fault and moves to the fault state. As IMU-2 does not return to an operational state, the SFDI algorithm persists in the same condition until the end of simulation. A slow drift is difficult to detect and isolate, due to the small slope, causing a delay between the beginning of the fault and the isolation. However, the correction of the magnetometer and the use of the average attitude (green line) in the normal state limit the error in the yaw estimation of some degrees (<3 deg).

Figure 8b shows the norm of the estimated gyroscope biases for both IMUs. It is worth noting that the bias estimated by IMU-2 is increasing over time due to the fault, while IMU-1 is almost hidden by the x-axis.

#### 6.1.3. Scenario F3—Abrupt Freezing

**Figure 9:**
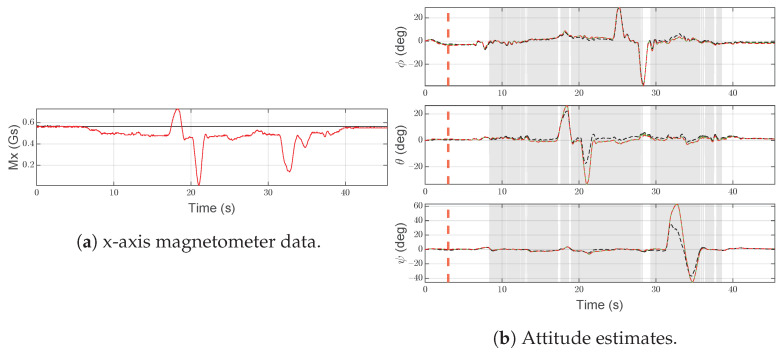
Scenario F3. Black lines denote measurements/estimates from IMU-1, red lines are for measurements/estimates from IMU-2. Green lines represent the output of the system, gray areas highlight the time interval in which the SFDI algorithm is in the fault state. Vertical red dashed lines denote the time instants when the fault occurs and ends.

Figure 9a shows *x*-axis magnetometer data $M_{x}^{\left(1\right)}$ from IMU-1 subject to an abrupt freezing over almost the whole simulation.

Results in Figure 9b show that the SFDI algorithm switches between normal and fault states several times, when the broken value of $M_{x}^{\left(1\right)}$ is similar to the correct one and estimated attitudes are in the predefined threshold. Furthermore, it is worth noting that the fault was isolated with a significant delay. This behavior depends on magnetometer signals $M_{x}^{\left(1\right)}$ and $M_{x}^{\left(2\right)}$ that, in the first phases of the flight, are almost the same. At the take-off, $M_{x}^{\left(2\right)}$ is affected by a small change due to the magnetic field of the electric motors that is altered by an increasing power request. Because the faulted IMU is frozen, only at the take-off is the SFDI algorithm able to detect the fault. After that, at several times during the flight, both errors on attitudes and magnetometer signals are below the predefined threshold, causing the return to the normal state. However, the reliability of the overall system is not compromised because, as shown in Figure 9b, a sensor fault does not affect attitude estimation for a relevant time interval.

#### 6.1.4. Scenario F4—Oscillation

**Figure 10:**
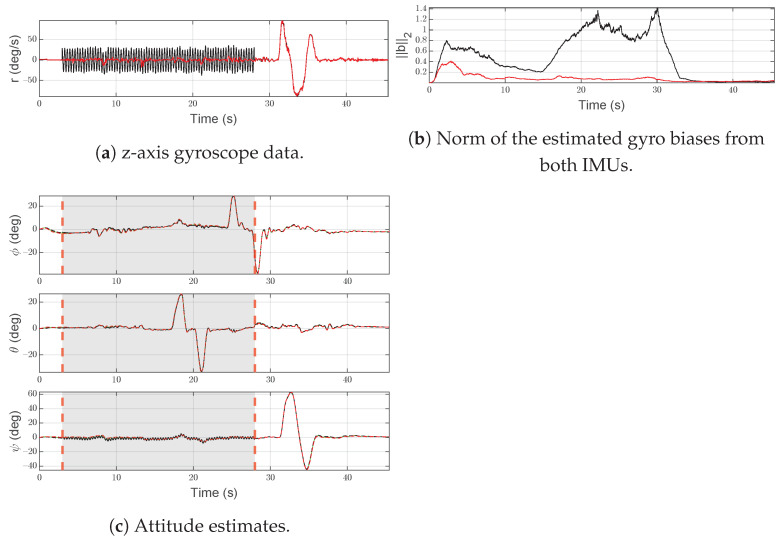
Scenario F4. Black lines denote measurements/estimates from IMU-1, red lines are for measurements/estimates from IMU-2. Green lines represent the output of the system, gray areas highlight the time interval in which the SFDI algorithm is in the fault state. Vertical red dashed lines denote the time instants when the fault occurs and ends.

In Figure 10a, the considered fault on the *z*-axis gyroscope measurement $r_{S}^{\left(1\right)}$ is shown. In particular, a sinusoidal disturbance, with a frequency (∼10 Hz), compliant to tri-rotor dynamics, is added to $r_{S}^{\left(1\right)}$ in the time interval t∈[3,28] s.

As shown in Figure 10c, although the attitude estimated by IMU-1 is only partially affected by the fault, the SFDI algorithm is able to detect the problem after a very short time, with the transition to the fault state from t=3.02 s to t=28.01 s denoting a fast return to the normal state, once the signal on IMU-1 becomes operational.

In Figure 10b, the norm of the estimated gyro biases is shown, highlighting the difference between IMUs.

#### 6.1.5. Scenario F5—Random Walk

**Figure 11:**
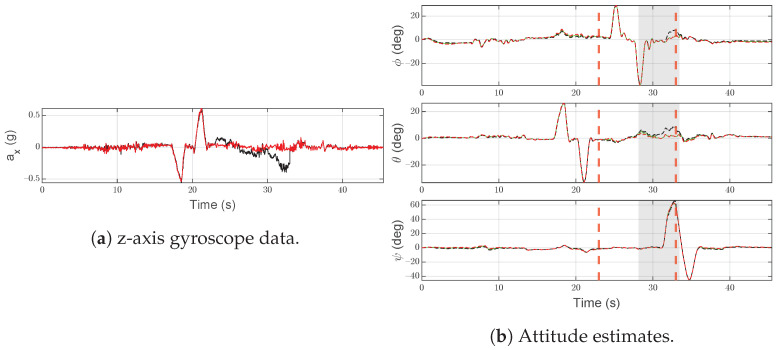
Scenario F5. Black lines denote measurements/estimates from IMU-1, red lines are for measurements/estimates from IMU-2. Green lines represent the output of the system, gray areas highlight the time interval in which the SFDI algorithm is in the fault state. Vertical red dashed lines denote the time instants when the fault occurs and ends.

In Figure 11a, the effects of the random walk disturbance on accelerometer data are shown. This signal is created by integrating a Gaussian noise with a mean value of 0.07 g and overlapped to the real signal in the time interval [23 s, 33 s]. In order to stress the algorithm, the mean value of noise is lower than the relevant detection threshold.

As shown in Figure 11b, the SFDI algorithm moves to the fault state at t=28.16 s, with a notable delay from the beginning of the injected fault. The reliability of the estimated attitude is not affected and such delay depends on the tri-rotor attitude: without any relevant maneuver affecting a change in the pitch angle, a small error on $a_{x,S}^{\left(1\right)}\left(t\right)$ results in a small trace on the attitude (<1 deg in the simulation). After IMU-1 becomes newly operational, the SFDI algorithm returns to the normal state (at t=33.41 s).

### 6.2. Algorithm Parameter Sensitivity Analysis

Effects of parameters on the performance of the proposed algorithm are discussed by means of a comparison between several choices. Scenario F1 is considered. We denote with $T_{0}=\{300,301,\cdots ,1300\}$ the discrete time set in which the fault is injected. Executing the SFDI algorithm the fault is detected and isolated. With $T_{f}=\left\{k:F_{1}\left(k\right)=1\right\}$ being the set of time instants in which IMU-1 is considered faulted, the following performance indices were considered:

1. *computation time*—the time needed to execute the SFDI algorithm at each time instant on the embedded platform Raspberry Pi 3B;
2. *correct detection*—the ratio between the correctly detected fault time interval and the real fault duration, computed as $CD=\mathrm{card}\left(T_{f}\cap T_{0}\right)/\mathrm{card}\left(T_{0}\right)$, where card(T) denotes the cardinality of set T;
3. *wrong detection*—the ratio between the badly detected fault time interval and the real fault duration, computed as $WD=\mathrm{card}\left(T_{f}\cap {\bar{T}}_{0}\right)/\mathrm{card}\left(T_{0}\right)$, where ${\bar{T}}_{0}$ is the complementary set of $T_{0}$;
4. *detection time*—the delay between the beginning of the fault and the detection, computed as $DT=(\min T_{f}-\min T_{0})$;
5. *recovery time*—the delay between the end of the fault and the fault recovery, computed as $RT=(\max T_{f}-\max T_{0})$.

In Table 3, the computing time is shown considering several numbers of particles Δ. Results are taken from 30 executions of the algorithm for each Δ value on the above mentioned Raspberry Pi 3B platform.

In Table 4, the effects in terms of variation of 2–5 performance indices for varying numbers of particles are shown. A growth in the number of particles decreases PF convergence time thus leading to a quicker recovery from a fault. However, if the output of both (operational and faulty) IMUs is close, an excessively short convergence speed may cause the mislabeling of a faulty IMU as operational, reducing the *correct detection* ratio. For these reasons increasing the Δ value behind a certain threshold brings no advantages, while increasing computational effort, see Table 3. In view of the sensitivity analysis results, the chosen value of Δ is boldfaced. It represents the number of particles, among the considered values, showing the best correct detection ratio while guaranteeing real-time execution.

In Table 5 the effects of $\tau_{a}$ are evaluated. As expected, reducing the fault detection threshold may increase the number of wrong detections, whereas high threshold values may increase the number of missed detections. Detection time appears to be zero with lower $\tau_{a}$ values because of false positives and the fact that recovery time may be affected by false detections. The chosen value of $\tau_{a}$ is boldfaced and it represents the trade-off, among the considered thresholds, which shows the best ratio between the *correct detection* index and the *wrong identification* one.

In Table 6 the influence of Ω is shown. A higher recovery threshold value leads to a shorter recovery time, which eventually leads to exiting the fault condition too early; it causes an increase in the number of missed alarms, reducing SFDI algorithm sensitivity. The chosen value of Ω is boldfaced and it represents the best threshold value in terms of acceptable recovery time.

Finally, in Table 7 the effect of the v parameter is evaluated. It affects PF convergence time in two opposite ways. Larger v values may boost the convergence speed by enlarging the region where the algorithm searches for the best state estimate, but it can also reduce the correct match ratio in case of faults whose magnitude is close to the relevant detection threshold. Neither the wrong identification ratio nor the detection time are influenced by v value. The chosen value of v is boldfaced and it represents the trade-off in terms of the ratio between the *correct detection* and the *wrong identification* indices.

## 7. Conclusions

In commercial unmanned aviation, an increase in the system reliability is needed in order to make flight risks acceptable.

With this aim, in this paper, the implementation of a sensor fault detection and isolation technique on low-cost hardware typical of commercial micro- and mini-UAVs was performed. Given the crucial role of attitude estimation in flying platform stabilization, the proposed procedure is aimed at detecting and isolating faults on inertial measurement units used to estimate UAV attitude. The hardware is based on a duplex architecture with two inertial measurement units (IMUs) including 3-axis gyroscopes, accelerometers and magnetometers. A particle filtering approach is proposed to deal with the attitude estimation problem, being able to deal with non-linear motion models and non-Gaussian noise distributions.

Results show the effectiveness of such an approach. Data gathered from a real flight of a tri-rotor flying platform were useful to test the SFDI algorithm with real sensor disturbances due to vibrations and changes to magnetic field. The proposed algorithm correctly detected every injected fault, including for challenging scenarios such as freezing faults and slow drifts.

The proposed architecture increases system reliability by doubling the number of on-board sensors. This produces a reduction in weight, costs and power consumption, in comparison to the gold standard of triplex architecture for avionics at the price of lower speed and accuracy.

In the literature, few duplex IMU architectures have been proposed. In Table 8, the authors compared the proposed SFDI algorithm with the SFDI logic presented in a previous work [72], to highlight the pros and cons of the current solution by comparing platforms in terms of detection time ($t_{d}$) and maximum attitude error ($e_{max}$).

The implementation of the algorithm on a Raspberry Pi low-cost embedded platform with two IMU boards has shown that, although a particle filter is considered a heavy algorithm, the proposed procedure can be used on low-cost unmanned aircraft, with an increase in system reliability and without a significant change in the overall cost.

To make the algorithm readily available and easily tunable, a sensitivity analysis of algorithm parameters on the SFDI performance was conducted, highlighting the chosen settings.

For the latest low-cost drones equipped with sonar, lidar and optical flow sensors, the future applications of our procedure are currently under research, using more sensors and also validating duplex heterogeneous architectures, where redundant sensor platforms can contain different sensors.

## Figures and Tables

**Table 1:** MEMS sensor characteristics from datasheets.

Name	MPU6050 Acc.	MPU6050 Gyro	HMC5883l Mag.
Dynamic range	±4 g	±500 deg s${}^{-1}$	±2.5 Gs
Digital resolution	0.122 mg	0.015 deg s${}^{-1}$	2.095 mGs
Total RMS noise	n.d.	0.05 deg s${}^{-1}$	n.d
Noise density	400gHz${}^{-2}$	0.005 deg s${}^{-1}$ Hz${}^{-2}$	n.d.
Noise floor	n.d.	n.d.	0.002 mGs

**Table 2:** Parameters used in numerical results ($I_{4\times 4}$ represents the identity matrix of size 4×4).

Parameter	Value
Δ	4096
v	${\left[\begin{array}{cccccc} 0.0633 & 0.3291 & 0.5291 & 0.2658 & 0.2658 & 0.2658 \end{array}\right]}^{T}$
$\tau_{a}$	0.2411
$\tau_{g}$	0.2853
$\tau_{m}$	0.0591
Ω	$24.213\cdot I_{4\times 4}$

**Table 3:** Raspberry Pi computing time for several numbers of particles (Δ).

Number of Particles	Computation Time (s)	
	Average	Standard Deviation
1024	0.0138	0.0012
2048	0.0303	0.0022
**4096**	**0.0682**	**0.0030**
8192	0.1573	0.0050
16,384	0.3401	0.0071

**Table 4:** Sensitivity analysis on Δ.

Number of Particles	Correct Detection (%)	Wrong Detection (%)	Detection Time (s)	Recovery Time (s)
1024	89.81	0.00	0.27	11.56
**4096**	**99.50**	**0.00**	**0.27**	**6.96**
16,384	92.81	0.30	0.27	4.07
32,768	92.81	0.00	0.27	4.04

**Table 5:** Sensitivity analysis for varying of $\tau_{a}$.

$\alpha \cdot \tau_{a}$	Correct Detection (%)	Wrong Detection (%)	Detection Time (s)	Recovery Time (s)
α=0.25	99.40	24.36	0.00	18.67
α=0.75	99.90	0.06	0.00	6.96
α=1	**99.50**	**0.00**	**0.27**	**6.96**
α=1.5	45.75	1.03	0.27	6.96

**Table 6:** Sensitivity analysis for varying of Ω.

α·Ω	Correct Detection (%)	Wrong Detection (%)	Detection Time (s)	Recovery Time (s)
α=0.25	97.30	0.00	0.27	10.28
α=0.5	97.30	0.00	0.27	7.61
α=0.75	97.30	0.00	0.27	7.44
α=1	**99.50**	**0.00**	**0.27**	**6.96**
α=4	35.26	0.84	0.27	0.00

**Table 7:** Sensitivity analysis for varying of v.

α·v	Correct Detection (%)	Wrong Detection (%)	Detection Time (s)	Recovery Time(s)
α=0.25	88.11	0.00	0.27	32.45
α=0.5	85.71	0.00	0.27	4.43
α=0.75	43.36	0.00	0.27	0.00
α=1	**99.50**	**0.00**	**0.27**	**6.96**
α=4	94.31	0.00	0.27	4.08

**Table 8:** Comparison between duplex architectures.

	PF + SFDI		Previous Work [72]	
X	**$t_{d}$ (s)**	**$e_{\max}$ (rad)**	**$t_{d}$ (s)**	**$e_{\max}$ (rad)**
F1	0.270	0.018 (on ϕ angle)	0.094	0.073 (on ϕ angle)
F2	0.960	0.024 (on ψ angle)	2.075	0.026 (on ψ angle)

## Data Availability

The data presented in this study are available on request from the corresponding author.
